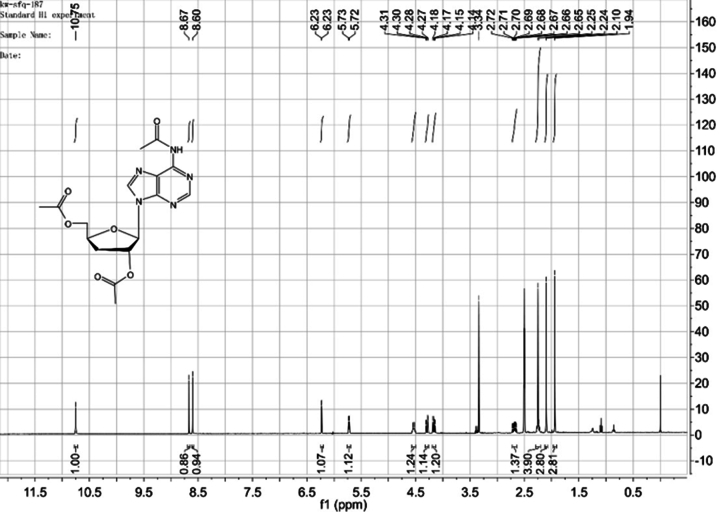# The effects of acetylated cordycepin derivatives on promoting vascular angiogenesis and attenuating myocardial ischemic injury

**DOI:** 10.1016/j.heliyon.2024.e40026

**Published:** 2024-11-01

**Authors:** Tzu-Ching Chang, Chao-Feng Lin, Yi-Jhu Lu, Shu-Man Liang, Jia-Yi Wei, Chih-Hui Chin, Song-Kun Shyue, Cheng-Chin Kuo, Jun-Yang Liou

**Affiliations:** a Institute of Cellular and System Medicine, National Health Research Institutes, Zhunan, Taiwan; b Department of Medicine, MacKay Medical College, New Taipei City, Taiwan; c Division of Cardiology, Department of Internal Medicine, MacKay Memorial Hospital, Taipei, Taiwan; d Cardiovascular Center, Cathay General Hospital, Taipei, Taiwan; e School of Medicine, Fu Jen Catholic University, New Taipei City, Taiwan; f Institute of Biomedical Sciences, Academia Sinica, Taipei, Taiwan; g Graduate Institute of Biomedical Sciences, China Medical University, Taichung, Taiwan

**Keywords:** Acetylated derivatives, Angiogenesis, Cordycepin, Endothelial cells, Myocardial infarction

## Abstract

**Background:**

Enhanced angiogenesis following myocardial infarction (MI) is beneficial to preserve cardiac function. The present study aimed to investigate whether acetylated derivatives of cordycepin altered its original antitumor properties and exerted cardioprotective effects by promoting angiogenesis *in vitro* and *in vivo*.

**Methods:**

Cordycepin and its derivatives with single (DA), double (DAA), and triple acetyl groups (DAAA) were assessed. The cell viability of leukemia U937 cells, malignant hepatoma Huh-7 cells, and human umbilical vascular endothelial cells (HUVECs) treated with cordycepin, DA, DAA, and DAAA were determined. The expression of β-catenin in U937 cells, as well as the expression of p65, p38 and other related signal regulators in HUVECs elicited by lipopolysaccharides (LPS) were also observed. Angiogenesis was determined by tube formation in HUVECs and Matrigel plug assay in mice. Cardiac function following administration of DAAA was evaluated in mice MI model simulated by coronary artery ligation.

**Results:**

The inhibitory effects of cordycepin and its acetylated derivatives on U937 cells, Huh-7 cells, HUVECs, and the expression of β-catenin in U937 cells were mitigated with increasing acetylation. Intriguingly, DAAA preserved the cell viability of HUVECs compared to other acetylated derivatives. Although DAAA had a significantly diminished antitumor effect compared to cordycepin, it promoted angiogenesis in mice and tube formation in HUVECs and attenuated LPS-induced phosphorylation of p65 and p38. Additionally, administration of DAAA improved cardiac function following coronary artery ligation in mice.

**Conclusion:**

DAAA could be considered a promising adjunctive therapy to prevent post-MI heart failure through promoting angiogenesis.

## Graphical abstract

**Figure:**
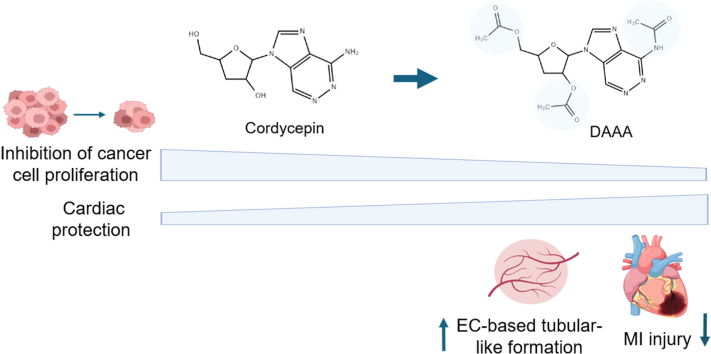

## Abbreviations:

MTT =: 3-(4,5-Dimethylthiazol-2-yl)-2,5-diphenyltetrazolium bromide
DAA =: 3’-Deoxy-2’,5’-di-O-acetyladenosine
DA =: 3’-Deoxy-5’-O-acetyladenosine
DAAA =: 3’-Deoxy-6-N-acetyl-2’,5’-di-O-acetyladenosine
DMSO =: dimethyl sulfoxide
EC =: endothelial cell
FBS =: fetal bovine serum
HCC =: hepatocellular carcinoma
HUVECs =: Human umbilical vein endothelial cells
LAD =: left anterior descending coronary artery
LV =: left ventricle
LVEF =: left ventricular ejection fraction (LVEF)
LPS =: lipopolysaccharides
MI =: myocardial infarction

## 1 Introduction

Despite implementation of current evidence-based treatments, myocardial infarction (MI) remains a major cause of reduced left ventricular (LV) function and post-MI heart failure [[1], [2], [3]]. It has been demonstrated that enhanced angiogenesis following MI may salvage cardiomyocytes from cell death and alleviate adverse cardiac remodeling [4,5], indicating that pharmacological therapy aimed at promoting angiogenesis should be considered as an important strategy in prevention of post-MI cardiac dysfunction.

**Fig. 1:**
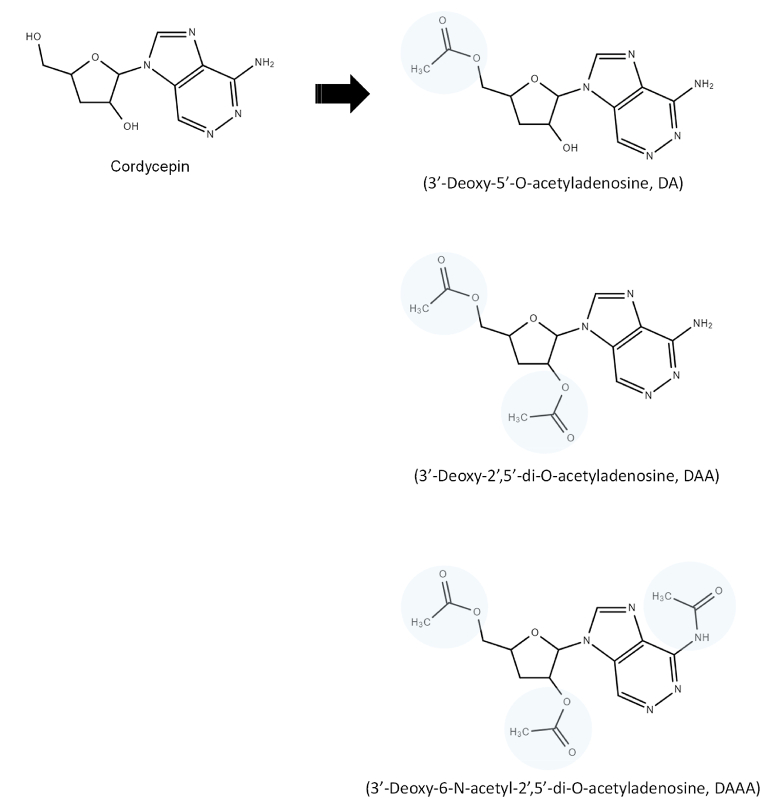
Chemical structure acetylated derivatives of cordycepin.

Cordycepin (also known as 3-deoxyadenosine), an active ingredient found in the fungus-infected caterpillars of *Cordyceps militaris* (Fig. 1), has been initially shown to exhibit antitumor properties in various types of cancers [6,7], including hematopoietic and solid malignancies. For example, cordycepin suppresses proliferation of leukemia cells via regulation of glycogen synthase kinase 3β and β-catenin signaling [6]. Furthermore, cordycepin may be utilized as an adjunctive therapy for hepatocellular carcinoma (HCC) by inhibiting integrin/focal adhesion kinase signaling [7]. In addition to antitumor effects, previous studies also demonstrated that cordycepin might be beneficial in cardiovascular diseases [8,9]. In cardiomyocytes cultured in a stress circumstance with high-glucose and high-fat medium, cordycepin prevented cardiomyocytes from apoptosis by enhancing mitofusin 2-medicated mitochondrial function [8]. Furthermore, cordycepin attenuated cardiac hypertrophy in mice presenting with LV pressure overload caused by aortic banding [9]. These novel findings drive further in-depth investigation focusing on cordycepin and its derivatives, particularly within the scope of antitumor and cardiovascular diseases.

Despite the fact that cordycepin exhibits antitumor and cardioprotective effects, it still remains unknown whether functional group modification of cordycepin would lead to alteration of its biomedical properties. To address this knowledge gap, the present study aimed to investigate the antitumor and angiogenic effects of cordycepin’s acetylated derivatives *in vitro* and *in vivo*. Ultimately, the results of our extensive experimentation confirmed acetylation to affect the original properties of cordycepin, particularly in regards to antitumor and cardioprotective effects.

## 2 Materials and methods

### 2.1 Cell culture and reagents

Human umbilical vein endothelial cells (HUVECs) were purchased from the American Type Culture Collection (Manassas, VA, USA) and were maintained in EGM-2 medium containing growth factors (Clonetics), 100 IU/mL of penicillin G sodium, 100 μg/mL of streptomycin sulfate, and 10 % (v/v) fetal bovine serum (FBS) at 37 °C in 5 % CO_2_/95 % air humidified atmosphere. Culture flasks were precoated with gelatin 0.2 % in ultra-purified water for 15 min and were dried for 6 h in a sterile environment. The U937 cell line was cultured in RPMI medium, and HCC cell line Huh-7 (Japanese Collection of Research Bioresources, Osaka, Japan) was cultured in Dulbecco’s modified Eagle’s medium supplemented with 10 % FBS (Hyclone; Thermo Fisher Scientific, Waltham, MA, USA). The chemical synthesis of acetylated derivatives of cordycepin involved several steps starting with 3'-deoxyadenosine. The detailed procedure is as follows: 3'-Deoxyadenosine served as the initial material for synthesizing 3’-Deoxy-6-N-acetyl-2’,5’-di-O-acetyladenosine (DAAA). The synthesis commenced by dissolving 3'-deoxyadenosine in absolute dimethylformamide, which was then cautiously added dropwise to acetic anhydride at 0 °C for 1 h. Subsequently, a Lewis base, N,N-Diisopropylethylamine, was slowly added, and the mixture was refluxed at 80 °C with stirring for 8 h. The resulting reaction mixture, containing 3’-Deoxy-5’-O-acetyladenosine (DA), 3’-Deoxy-2’,5’-di-O-acetyladenosine (DAA), and DAAA, was cooled to room temperature. After solvent removal by filtration or under vacuum, the residue was further analyzed using thin layer chromatography to separate the reacted products and obtain purified DA, DAA, and DAAA. The chemical structures of the three synthetic compounds were determined by both LC-MS and proton NMR spectroscopy (Fig. 1).

### 2.2 Cell viability analysis

Cell viability was analyzed by 3-(4,5-Dimethylthiazol-2-yl)-2,5-diphenyltetrazolium bromide (MTT) assay. Cells were seeded into 96-well plates and incubated with RPMI medium (Hyclone; Thermo Fisher Scientific, Waltham, MA, USA) containing cordycepin, DA, DAA, and DAAA with the indicated concentrations and incubation time as per the protocol in the present study. MTT was added to each well and incubated at 37 °C for 3 h. Subsequently, the yellow MTT solution was removed, and 200 μL of dimethyl sulfoxide (DMSO) was added. The absorbance at 570 nm was then measured with a reference wavelength of 690 nm.

### 2.3 Western blot analysis

Briefly, cells treated by cordycepin, DA, DAA, and DAAA at the indicated concentrations and incubation time were transferred to RIPA lysis buffer (Millipore, Billerica, MA, USA), containing protease and phosphatase inhibitor cocktails. Supernatant of cell lysate was collected and 30 μg of total protein from each sample then loaded onto a gradient SDS-PAGE gel, which were subsequently transferred to PVDF membranes. The membranes were blocked and hybridized with primary antibodies against β-catenin, phosphorylate ERK (p-ERK), phosphorylated p65 (p-p65) at Serine^536^ (S536) position, phosphorylated p65 (p-p65) at Serine^276^ (S276) position, phosphorylated mTOR (p-mTOR), phosphorylated p38 (p-p38), phosphorylated Akt (p-Akt), ERK, p65, mTOR, p38, Akt, and β-actin. Then, the membranes were incubated with secondary antibodies, and immunoreactive bands were visualized by X-ray film exposure. Protein expression levels were quantified by densitometric analysis.

### 2.4 Tube formation assay

HUVECs (1.4 × 10^5^) were pretreated with or without DAAA (100 μM) in 10 % FBS medium for 48 h, re-seeded into 24-well plates pre-coated with 200 μL/well Matrigel (Corning, Bedford, MA, USA), and incubated at 37 °C for 30 min. Then, cells in the Matrigel-coated 24-well plates were incubated with M200 medium supplemented with 1 % FBS and DAAA. After incubation for 6 h, the tube formation of HUVECs was examined by counting the number of branches at every 5 random fields using a microscope.

### 2.5 Matrigel plug angiogenesis assay *in vivo*

All animal experimental protocols of the present study were approved by the Institutional Animal Care and Use Committee of the Academia Sinica (13-06-558), Taiwan. C57BL/6 mice were purchased from the National Laboratory Animal Center, Taiwan. Adult male C57BL/6 mice (8–10 weeks, weighing 20–25g) were implanted with 250 μL Matrigel (Corning) containing 100 ng/mL VEGF (Sigma-Aldrich) and 20 U/mL heparin (B. Braun Melsungen AG, Melsungen, Germany) with or without 25 μg/mL DAAA for 7 days. The level of hemoglobin was measured using Drabkin’s reagent (Sigma-Aldrich).

### 2.6 Mice MI model by coronary artery ligation

Adult male C57BL/6 mice (8–10 weeks, weighing 20–25 g) were obtained from the National Laboratory Animal Center, Taiwan. Mice were cared for with living conditions of constant temperature and a 12-h light cycle. Mice MI model was established by coronary artery ligation. Briefly, under anesthesia with intraperitoneal injection of pentobarbital sodium (50 mg/kg), animals were intubated and ventilated with a small animal ventilator (Harvard Apparatus, Holliston, MA, USA). After thoracotomy, the left anterior descending (LAD) coronary artery was ligated by 7–0 silk sutures. Each mouse was subjected to permanent ligation and MI. After ligation, thoracic muscle and skin were closed by suture carefully, then endotracheal tubes and ventilator were removed. Mice were transferred to a single cage for further usual care. In our *in vivo* experiment, mice were randomized into 3 groups: (1) Sham group: mice underwent the surgery procedure, but without induction of MI; (2) MI group: mice received MI surgery with administration of DMSO; and (3) MI + DAAA group: mice received MI surgery and administration of DAAA, which was given by implantation of intraperitoneal pumps with releasing dose of 15 mg/kg/day.

### 2.7 Echocardiographic analysis of cardiac function

LV systolic function was determined by LV ejection fraction (LVEF) with murine echocardiogram. Mice were anaesthetized with 1.5 % isoflurane. Transthoracic echocardiography was performed at 100 mm/s and 15 MHz linear transducer on an Acuson SequioaTM 256 system (Siemens Medical, CA, USA). LVEF of each animal was calculated with manual mapping of circumferential systolic and diastolic LV chamber areas in a two-dimensional (2D) model.

### 2.8 Statistics

The Student’s t-test was used to analyze differences between 2 experimental groups. A *p* value of less than 0.05 was considered statistically significant.

## 3 Results

### 3.1 The effects of cordycepin and its acetylated derivatives on cell viability of U937 cells

**Fig. 2:**
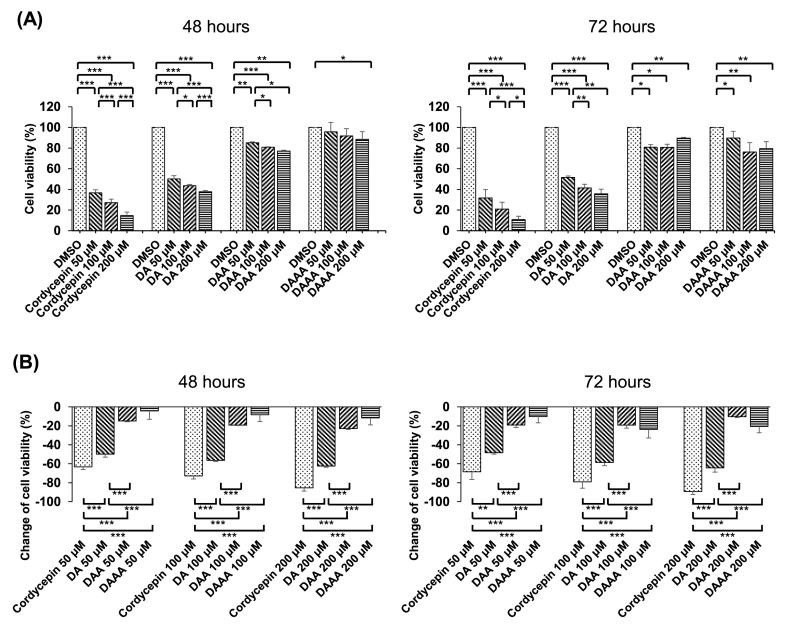
The effects of cordycepin and its acetylated derivatives at different doses (50, 100, and 200 μM) on cell viability of U937 cells. (A) Under the same dose of cordycepin and its acetylated derivatives, the cell viability of U937 cells increased with increasing acetylation at the incubation time of 48 h (left panel) and 72 h (right panel). (B) Under the same dose of cordycepin and its acetylated derivatives, the negative changes of cell viability of U937 cells diminished with increasing acetylation at the incubation time of 48 h (left panel) and 72 h (right panel). ∗*p* < 0.05, ∗∗*p* < 0.01, ∗∗∗*p* < 0.001.

Here we used the U937 cell line, a widely used cell model of leukemia [10,11], to determine the antitumor properties of cordycepin and its acetylated derivatives. The cell viability of U937 cells treated with cordycepin, DA, DAA, and DAAA at various concentrations (50, 100, and 200 μM) for 48 or 72 h was assessed by MTT assay (Fig. 2). We observed that U937 cells treated with cordycepin, DA, and DAA showed a significant reduced viability in a dose-dependent manner, irrespective of incubation time, compared to the cells treated with DMSO. However, the cell viability of U937 cells treated with DAAA, compared to the cells treated with DMSO, was not reduced until at a higher dose (200 μM) or with a longer incubation time (72 h) (Fig. 2A). The inhibitory effects of DA, DAA, and DAAA, calculated by the percentage change in cell viability of U937 cells, were obviously mitigated with increasing acetylation compared to cordycepin (Fig. 2B).

### 3.2 The effects of cordycepin and its acetylated derivatives on the expression of β-catenin in U937 cells

**Fig. 3:**
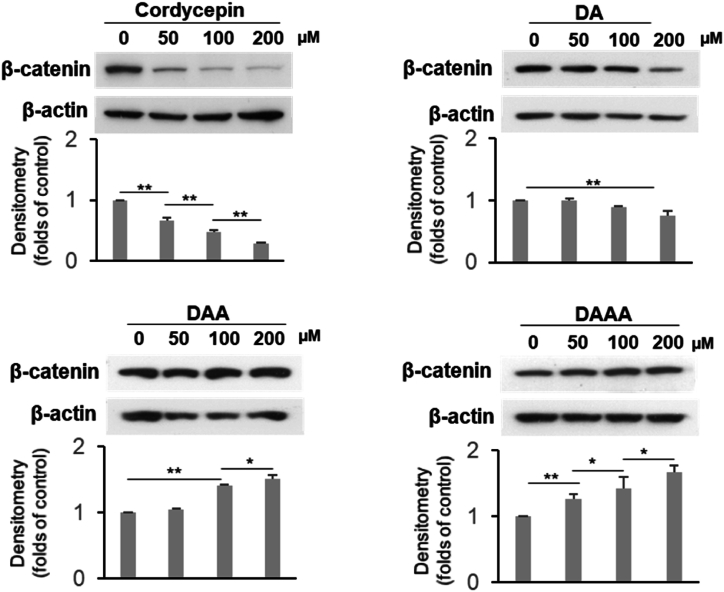
The effects of cordycepin and its acetylated derivatives on the expression of β-catenin in U937 cells. The expression of β-catenin in U937 cells were detected by Western blot analysis and quantified. ∗*p* < 0.05, ∗∗*p* < 0.01.

Because β-catenin has been shown to be a key factor in maintaining hepatocarcinogenesis [12] and clonogenic capacities of leukemia [13], we examined the expression of β-catenin in U937 cells incubated with cordycepin and its acetylated derivatives (Fig. 3). Cordycepin reduced the expression of β-catenin in U937 cells in a dose-dependent manner, as was also observed in our previous study [6]. DA had a similar effect as cordycepin in reducing the expression of β-catenin, while DAA and DAAA had no significant suppressive effects on the expression of β-catenin in U937 cells (Fig. 3). In contrast, DAA and DAAA at a high dose (100 or 200 μM) exhibited an enhanced effect on the expression of β-catenin in U937 cells (Fig. 3). These findings indicated that the antitumor effects of cordycepin were mitigated with increasing acetylation.

### 3.3 The effects of cordycepin and its acetylated derivatives on Huh-7 and HUVECs

**Fig. 4:**
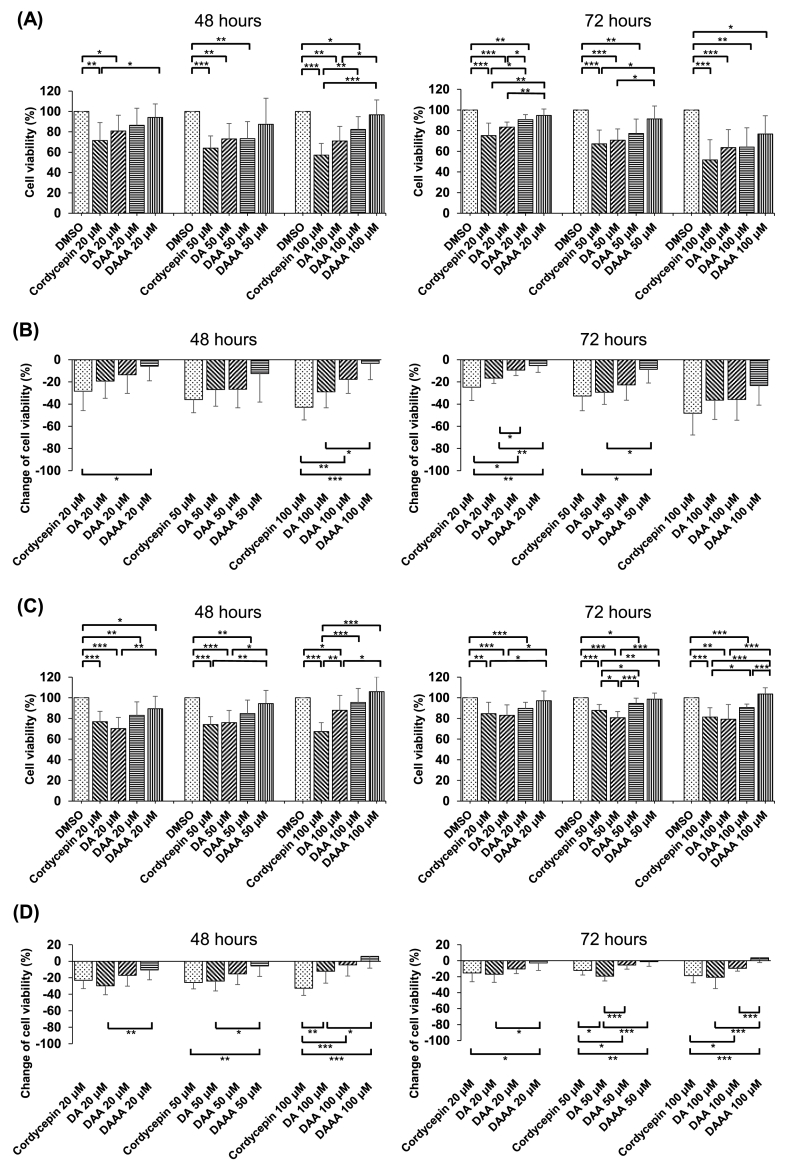
The effects of cordycepin and its acetylated derivatives at different doses (20, 50, and 100 μM) on Huh-7 and HUVECs. (A) Under the same dose of cordycepin and its acetylated derivatives, the cell viability of Huh-7 cells increased with increasing acetylation at the incubation time of 48 h (left panel) and 72 h (right panel). (B) Under the same dose of cordycepin and its acetylated derivatives, the negative changes of cell viability of Huh-7 cells diminished with increasing acetylation at the incubation time of 48 h (left panel) and 72 h (right panel). (C) Under the same dose of cordycepin and its acetylated derivatives, the cell viability of HUVECs increased with increasing acetylation at the incubation time of 48 h (left panel) and 72 h (right panel). (D) Under the same dose of cordycepin and its acetylated derivatives, the negative changes of cell viability of HUVECs diminished with increasing acetylation at the incubation time of 48 h (left panel) and 72 h (right panel). DAAA at 100 μM even promoted the cell viability of HUVECs. ∗*p* < 0.05, ∗∗*p* < 0.01, ∗∗∗*p* < 0.001.

Apart from the U937 leukemia model, we also applied cordycepin to other cell lines to determine cell viability. In the process, we discovered that the results of MTT assay in Huh-7 cells, a hepatocellular carcinoma cell line, were similar to those of U937 cells. The cordycepin derivatives exhibited a gradual decrease in their effects on inhibiting Huh-7 cells with increasing numbers of acetyl groups, irrespective of various incubation concentrations or time, in which DAAA had a significantly lower effect in inhibiting the cell viability of Huh-7 cells compared to cordycepin (Fig. 4A). The percentage change in Huh-7 cell viability was calculated to show that the inhibitory effects of DA, DAA, and DAAA were significantly mitigated with increasing acetylation in comparison to cordycepin (Fig. 4B).

Cordycepin, as well as DA and DAA, inhibited the cell viability of HUVECs in varying degrees; however, we found that the effects of DA and DAA in suppressing HUVECs were gradually diminished with increasing acetyl functional groups (Fig. 4C). A similar pattern change of cell viability was observed in U937 and Huh-7 cells (Fig. 4D). Interestingly, DAAA had little effects in reducing cell viability of HUVECs, whereas cell proliferation of HUVECs were slightly promoted at a concentration of 100 μM (Fig. 4D). Based on these findings, we then further assessed the effects of DAAA on angiogenesis *in vitro* and *in vivo*.

### 3.4 Cordycepin derivative DAAA enhanced angiogenesis *in vitro* and *in vivo*

**Fig. 5:**
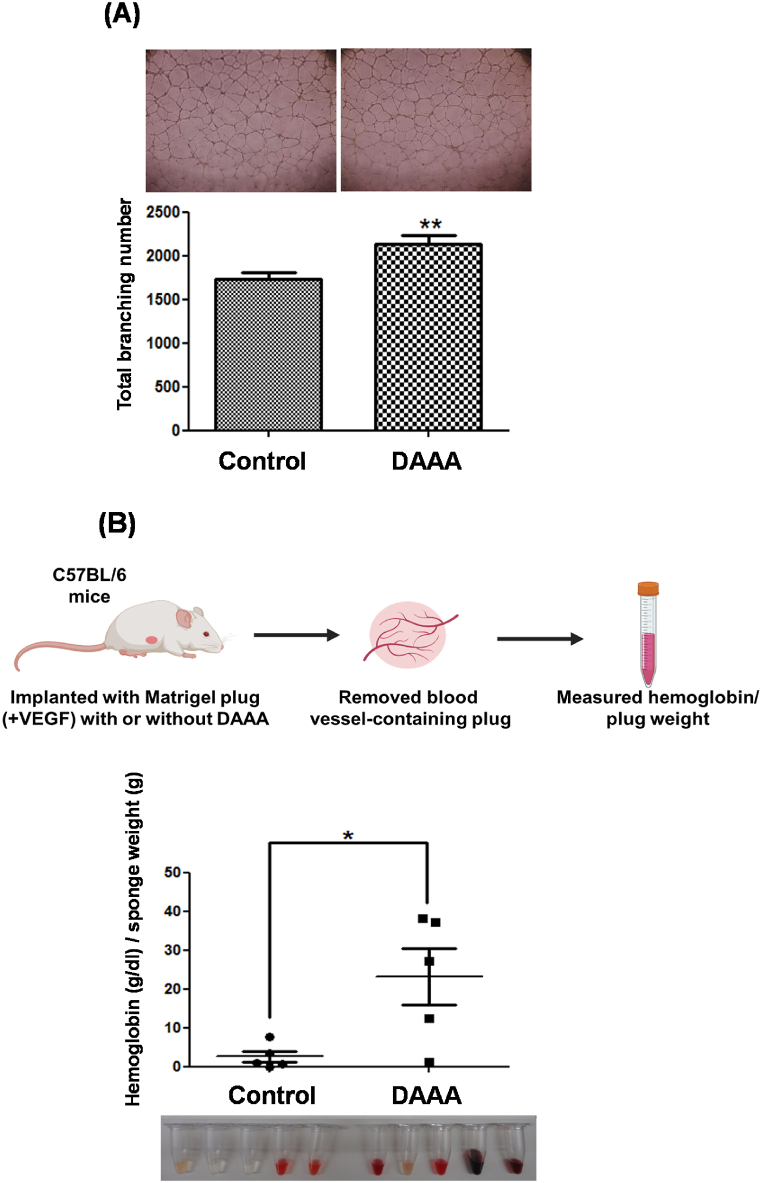
The effects of DAAA on angiogenesis *in vitro* and *in vivo*. (A) Incubation with DAAA (100 μM, 48 h) enhanced the tube formation of HUVECs. (B) DAAA increased hemoglobin contents determined by Matrigel plug assay. ∗*p* < 0.05, ∗∗*p* < 0.01.

To examine the effects of DAAA on angiogenesis, *in vitro* analysis of tube formation was performed. We found that the tube formation in HUVECs treated with DAAA was significantly higher compared to that of control HUVECs, which were treated with DMSO (Fig. 5A). In addition, the *in vivo* Matrigel plug angiogenesis assay showed that mice implanted with DAAA-containing plugs had a higher hemoglobin content compared to control mice, which were implanted with DMSO-containing plugs (Fig. 5B). These findings indicated that DAAA enhanced both *in vitro* and *in vivo* angiogenesis.

### 3.5 Effects of DAAA on regulation of various signal pathways

**Fig. 6:**
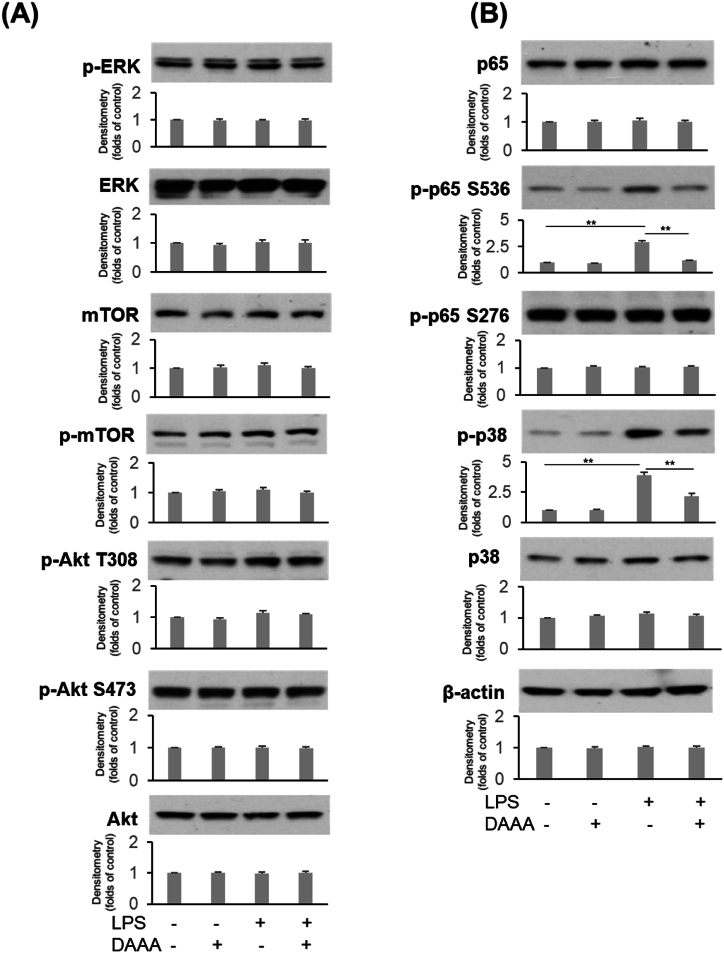
Effects of DAAA on regulation of various signal pathways. (A) Expression of ERK, mTOR, Akt and their phosphorylation treated with DAAA with/without LPS in HUVECs were determined by western blotting analysis. (B) Expression of NF-κB and p38 as well as their phosphorylation treated with DAAA with/without LPS in HUVECs were determined by western blotting analysis. Quantification of Western blot analysis was conducted by densitometry. ∗p < 0.05, ∗∗p < 0.01.

To elucidate the possible mechanisms of DAAA in promoting angiogenesis, several stress-induced angiogenic regulators and their phosphorylated forms, including ERK, mTOR, Akt, p65, and p38, were determined in HUVECs treated with LPS (Fig. 6). We observed that treatment with DAAA did not alter the expression of ERK, mTOR, and Akt, nor their phosphorylation induced by LPS in HUVECs (Fig. 6A). However, DAAA

reduced the LPS-induced phosphorylation of p65 at Serine^536^ (p-p65 S536) and p38 (p-p38) in HUVECs, whereas it did not alter the phosphorylation of p65 at Serine^276^ (p-p65 S276) (Fig. 6B).

### 3.6 DAAA restored cardiac function in mice model of myocardial infarction

**Fig. 7:**
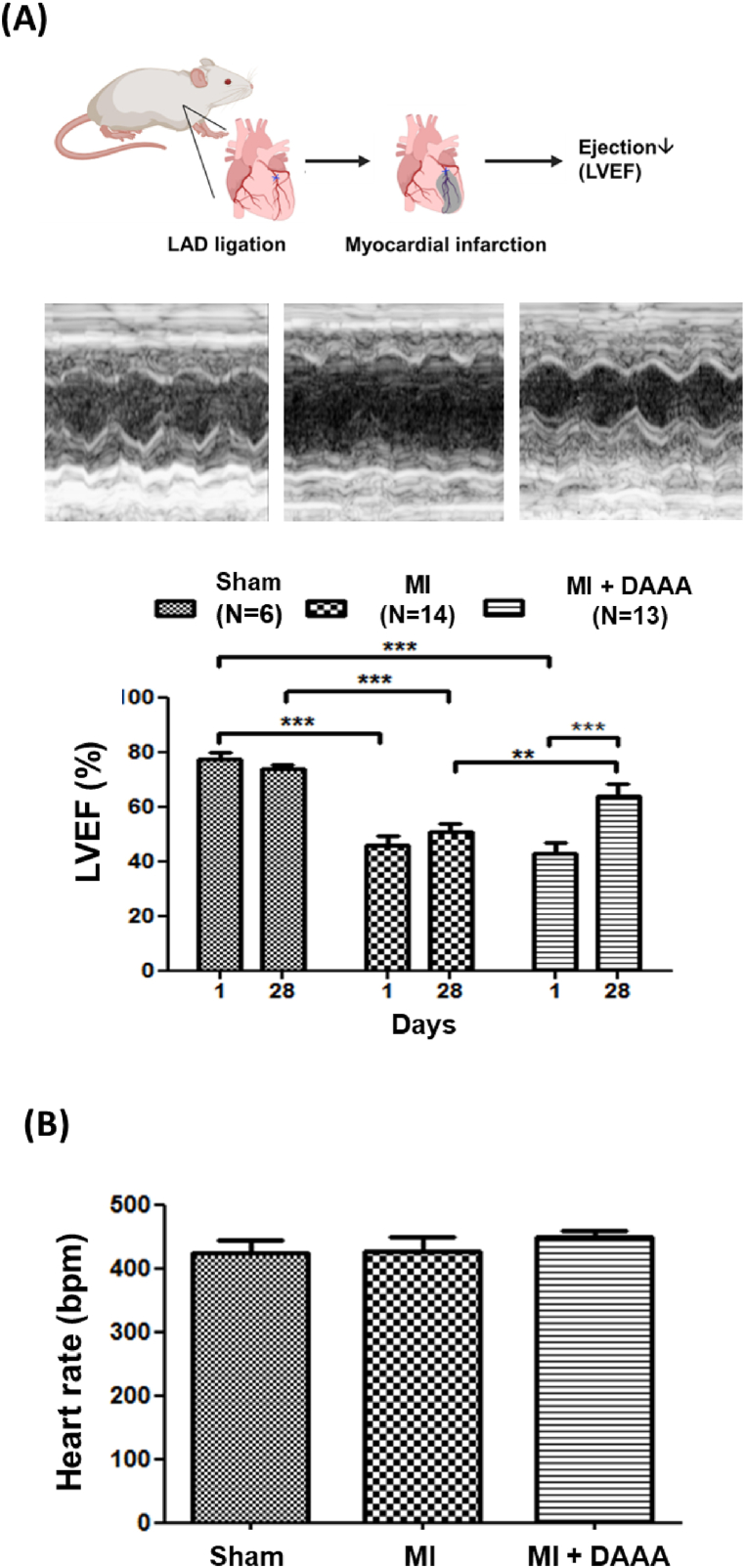
DAAA restored cardiac function in mice model of myocardial infarction. (A) LVEF of mice treated by DAAA was improved at the 28^th^ following MI. (B) Treatment of DAAA did not alter the heart rates of mice. ∗∗*p* < 0.01, ∗∗∗*p* < 0.001.

To assess the effects of DAAA on ischemic myocardial injury, an acute MI mouse model was applied, with treatment of DMSO or DAAA administered by implanted osmotic pumps. On day one after the surgery, mice that received coronary artery ligation had a significantly lower LVEF, as compared to mice that received sham surgery. Administration of DAAA significantly improved LVEF of mice at the 28^th^ day following coronary artery ligation compared to DMSO (Fig. 7A). Additionally, we also found that treatment of DAAA did not alter the heart rates of mice (Fig. 7B).

## 4 Discussion

Introducing acetyl functional groups into cordycepin may alter its original biomedical properties. Previous studies have investigated different derivatives of cordycepin with triple acetyl groups at distinct binding sites [14,15], revealing that these compounds possessed beneficial effects on alleviating cardiac hypertrophy and angiotensin II-induced cardiac fibrosis *in vivo* [14,15]. In line with these abovementioned studies, the present study also showed that DAAA, a unique acetylated derivative of cordycepin developed in our laboratory, exhibited a beneficial effect on promoting angiogenesis both *in vitro* and *in vivo*.

The main findings of our current study showed that the inhibitory effects of cordycepin on β-catenin were diminished with increasing acetylation, thereby alleviating the antitumor properties of acetylated derivatives. DAAA, a derivative of cordycepin with triple acetyl groups, showed a significant decrease in ability to suppress leukemia cells and HCC cells, instead exhibiting a pro-angiogenic trait through promotion of angiogenesis *in vitro* and *in vivo*. In the mice MI model, administration of DAAA demonstrated a favorable effect in improving LVEF without altering animals’ heart rates following MI, possibly through reducing the expression of stress-induced p-p65 (S536) and p-p38 in HUVECs. Our current study is the first report to investigate the pro-angiogenic effects of these unique acetylated derivatives.

Anti-angiogenesis has become a mainstream of cancer therapy; however, there is a growing awareness of an association between anti-angiogenic drugs and various ischemic cardiovascular complications, including MI, stroke, and cardiovascular death [16,17], implicating that the application of anti-angiogenic drugs in cardiovascular diseases should be cautiously performed. In contrast, drugs with pro-angiogenic properties via promoting endothelial cell (EC) function is beneficial in tissue repair after MI [5,18]. A properly coordinated angiogenesis is associated with favorable outcomes in animal MI models as evidenced by smaller infarct scars, less ventricular remodeling, and improved cardiac function [18]. In the present study, despite its diminished antitumor property, DAAA demonstrated a novel effect on enhancing angiogenesis, potentially making it a more suitable adjunctive therapy for MI, as compared to cordycepin.

After MI, the infiltration of neutrophils and macrophages derived from circulating monocytes elicits a robust immune response and various inflammation-related signals in ECs of the infarct border zone [[18], [19], [20]], including activation of NF-κB and p38 signal pathways [21,22]. Although phosphorylation of NF-κB subunit p65 at Serine^276^ is an essential contributor to NF-κB activation *in vitro* and *in vivo*, phosphorylation of NF-κB subunit p65 at Serine^536^ (p-p65 S536) represents the most potent position of phosphorylation [21,23], and plays a pivotal regulator in multiple inflammatory diseases. The expression of p-p65 S536 and p-p38 in ECs contributes to adhesion of immune cells, apoptosis of ECs, vascular hyperpermeability, and loss of endothelial integrity [22,24,25]. Moreover, phosphorylation of p38 in ECs has also been demonstrated to negatively regulate the tubular morphogenesis of ECs by decreasing the expression of differentiation-specific proteins, such as Notch ligand Jagged1 [26]. This evidence suggested possible mechanisms behind the enhanced angiogenesis induced by DAAA, which was observed to inhibit p-p65 S536 and p-p38 in HUVECs in our current study.

The substantial death of cardiomyocytes following MI leads to a detrimental remodeling process, including the formation of necrotic cavities and trabeculation within the endocardium and epicardium. These structural changes cause geometric alterations of cardiac chambers and a reduction in contractility [18,[27], [28], [29]]. Infarct healing with enhancing angiogenesis in myocardium may therefore present a window of therapeutic opportunity to prevent adverse ventricular remodeling and post-MI heart failure [3,18]. In mice MI model, endogenous angiogenesis is initiated within the infarct border zone by the formation of microvessels. The newly formed capillaries then undergo expansion, characterized by extensive branching and vessel sprouting into the infarct core, typically occurring between 2 and 4 days after coronary artery ligation [18,27]. Although the endogenous angiogenesis begins immediately after MI, it may still require up to 4 weeks to reestablish oxygenation in parallel with the formation of new vascular structures [30]. Therefore, the pharmacological strategies to enhance angiogenesis within 4 weeks following MI is beneficial, as supported by our findings, which show that administration of DAAA for 4 weeks exhibited a significant improvement in LVEF among MI mice. Further studies are warranted to investigate whether a longer duration of DAAA treatment shows more favorable outcomes. Collectively, our work provided valuable preclinical insights and contributed to the realm of drug discovery for addressing post-MI heart failure. Furthermore, our findings could be potentially applied to the treatment of other diseases, such as preeclampsia [31,32].

Our present study was subjected to some limitations. First, we did not determine the bioavailability of DA, DAA, and DAAA *in vivo* to evaluate their therapeutic efficiency and safety profiles. Second, the present study did not compare the effects between cordycepin and DAAA in mice MI model. Third, we only evaluated the effects of DAAA on ECs, so we could not observe the potential complex mechanisms of angiogenesis through interactions of ECs and other cell types. Future in-depth investigations are necessary to elucidate these issues, especially in regards to the histological analysis of heart tissue obtained from the mice MI experiments.

## 5 Conclusions

DAAA, a derivative of cordycepin with triple acetyl groups, exhibited a beneficial effect on enhancing *in vitro* and *in vivo* angiogenesis, potentially through inhibition of stress-induced phosphorylation of p65 S536 and p38. Additionally, administration of DAAA in animal MI model led to an improvement of cardiac function. Our findings highlight that DAAA should be considered as a promising therapeutic strategy to regulate angiogenesis and prevent heart failure following MI.

## CRediT authorship contribution statement

**Tzu-Ching Chang:** Writing – original draft, Formal analysis, Data curation, Conceptualization. **Chao-Feng Lin:** Writing – original draft, Formal analysis, Conceptualization. **Yi-Jhu Lu:** Formal analysis, Data curation. **Shu-Man Liang:** Formal analysis, Data curation. **Jia-Yi Wei:** Formal analysis, Data curation. **Chih-Hui Chin:** Writing – original draft, Formal analysis, Conceptualization. **Song-Kun Shyue:** Writing – original draft, Methodology, Formal analysis, Data curation, Conceptualization. **Cheng-Chin Kuo:** Writing – original draft, Resources, Methodology, Conceptualization. **Jun-Yang Liou:** Writing – review & editing, Writing – original draft, Supervision, Resources, Investigation, Funding acquisition, Formal analysis, Conceptualization.

## Data availability statement

Data will be made available on request.

## Funding

This study was supported by 10.13039/100020595National Science and Technology Council (108-2320-B-400-020-MY3 and 111-2320-B-400-013) and the 10.13039/501100004737National Health Research Institutes (10A1-CSPP07-014) of Taiwan.

## Declaration of competing interest

The authors declare that they have no known competing financial interests or personal relationships that could have appeared to influence the work reported in this paper.

## Figures and Tables

**Figure:**