# Supplementary material for: The effects of acetylated cordycepin derivatives on promoting vascular angiogenesis and attenuating myocardial ischemic injury
Source: Heliyon. 2024 Nov 1;10(21):e40026. doi: 10.1016/j.heliyon.2024.e40026 (PMC11567033; doi:10.1016/j.heliyon.2024.e40026)
Supplement: Multimedia component 1 [file mmc1.pptx]

## Slide 1
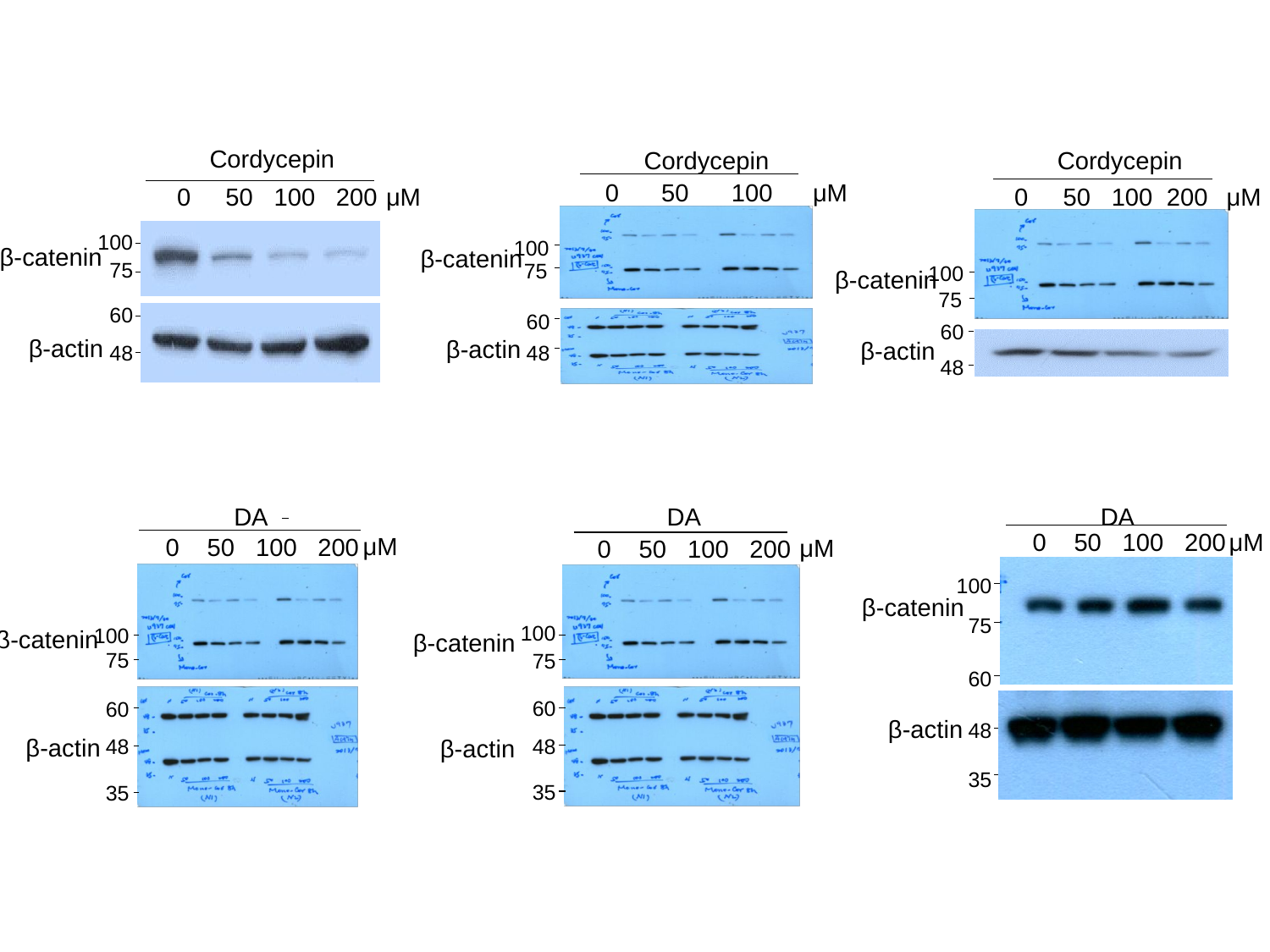

Cordycepin
 0 50 100 200
μM
100
75
60
48
β-catenin
β-actin
Cordycepin
 0 50 100 200
μM
100
β-catenin
75
60
β-actin
48
Cordycepin
 0 50 100 200
μM
100
β-catenin
75
60
β-actin
48
DA
μM
 0 50 100 200
100
75
60
48
35
β-catenin
β-actin
DA
μM
 0 50 100 200
100
β-catenin
75
60
β-actin
48
35
DA
μM
 0 50 100 200
100
75
60
48
35
β-catenin
β-actin

## Slide 2
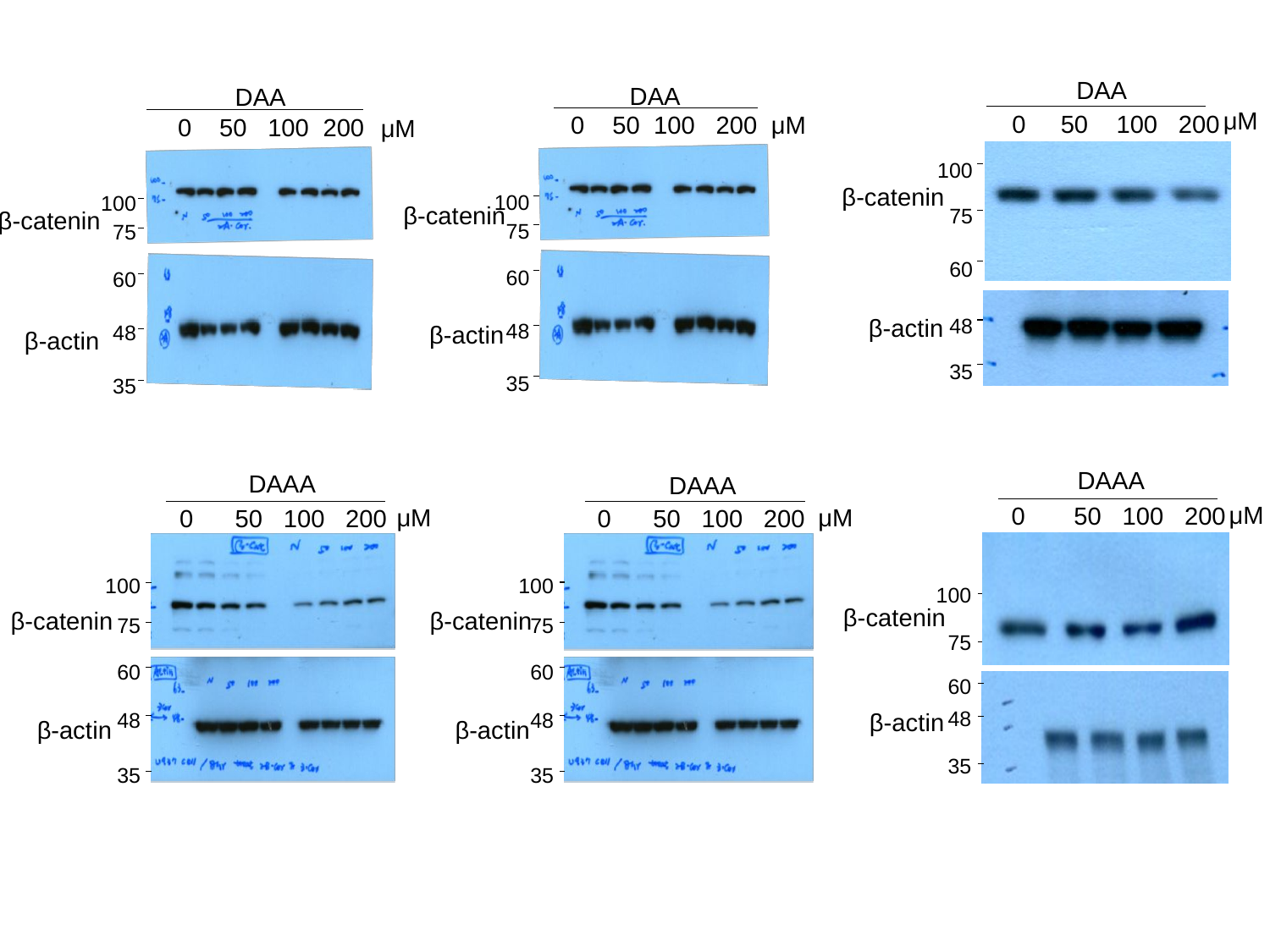

DAA
μM
 0 50 100 200
100
75
60
48
35
β-catenin
β-actin
DAA
μM
 0 50 100 200
100
75
60
48
35
β-catenin
β-actin
DAA
 0 50 100 200
μM
100
75
60
48
35
β-catenin
β-actin
DAAA
μM
 0 50 100 200
100
β-catenin
75
60
48
β-actin
35
DAAA
μM
 0 50 100 200
100
75
60
48
35
β-catenin
β-actin
DAAA
μM
 0 50 100 200
100
75
60
48
35
β-catenin
β-actin
