# Supplementary material for: The effects of acetylated cordycepin derivatives on promoting vascular angiogenesis and attenuating myocardial ischemic injury
Source: Heliyon. 2024 Nov 1;10(21):e40026. doi: 10.1016/j.heliyon.2024.e40026 (PMC11567033; doi:10.1016/j.heliyon.2024.e40026)
Supplement: Multimedia component 2 [file mmc2.pptx]

## Slide 1
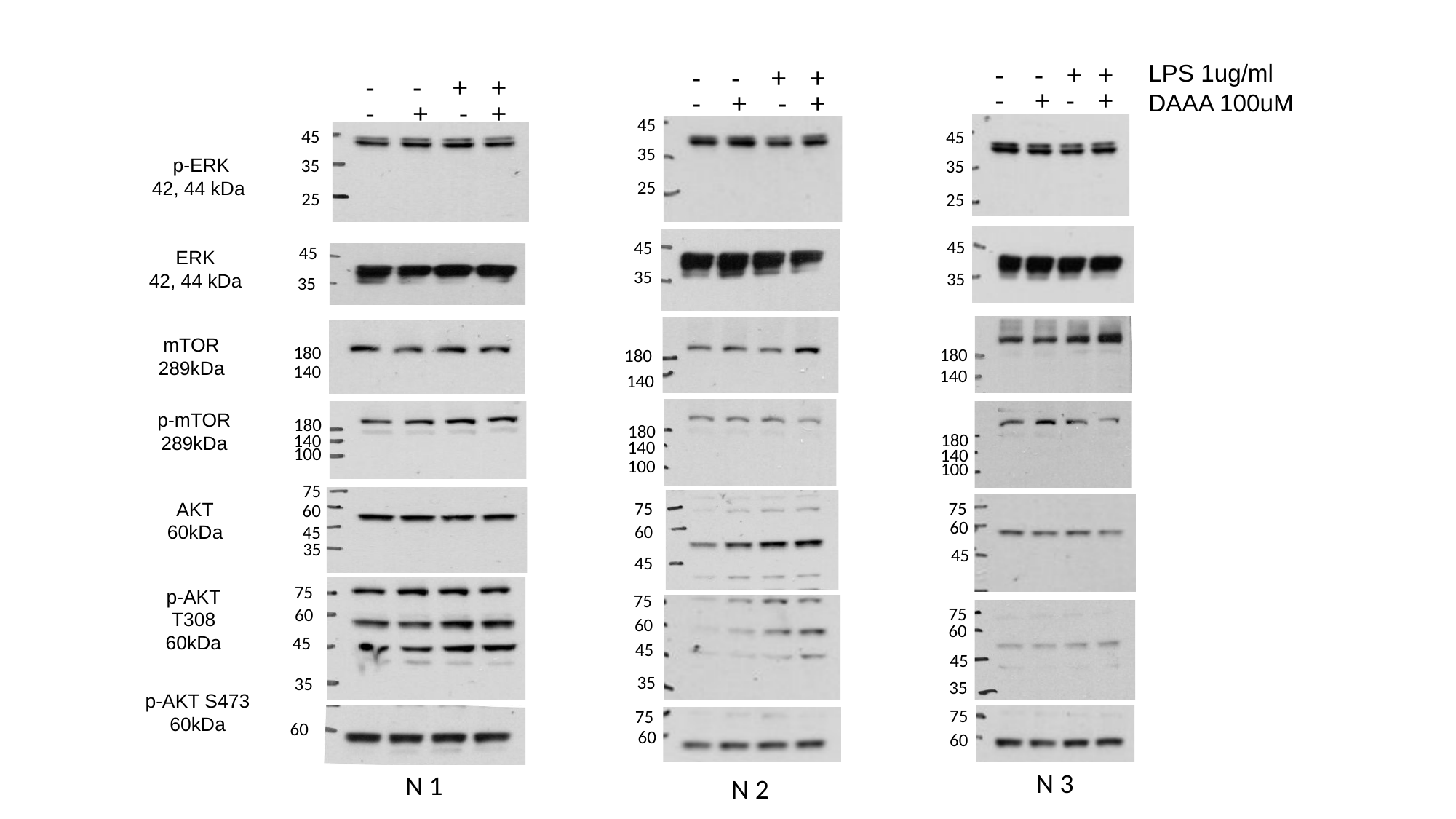

- - + +
LPS 1ug/ml
- - + +
- - + +
- + - +
- + - +
DAAA 100uM
- + - +
45
45
45
35
p-ERK
42, 44 kDa
35
35
25
25
25
45
45
45
ERK
42, 44 kDa
35
35
35
mTOR
289kDa
180
180
180
140
140
140
p-mTOR
289kDa
180
180
180
140
140
100
140
100
100
75
75
75
AKT
60kDa
60
60
60
45
35
45
45
75
p-AKT
T308
60kDa
75
75
60
60
60
45
45
45
35
35
35
p-AKT S473
60kDa
75
75
60
60
60
N 3
N 1
N 2

## Slide 2
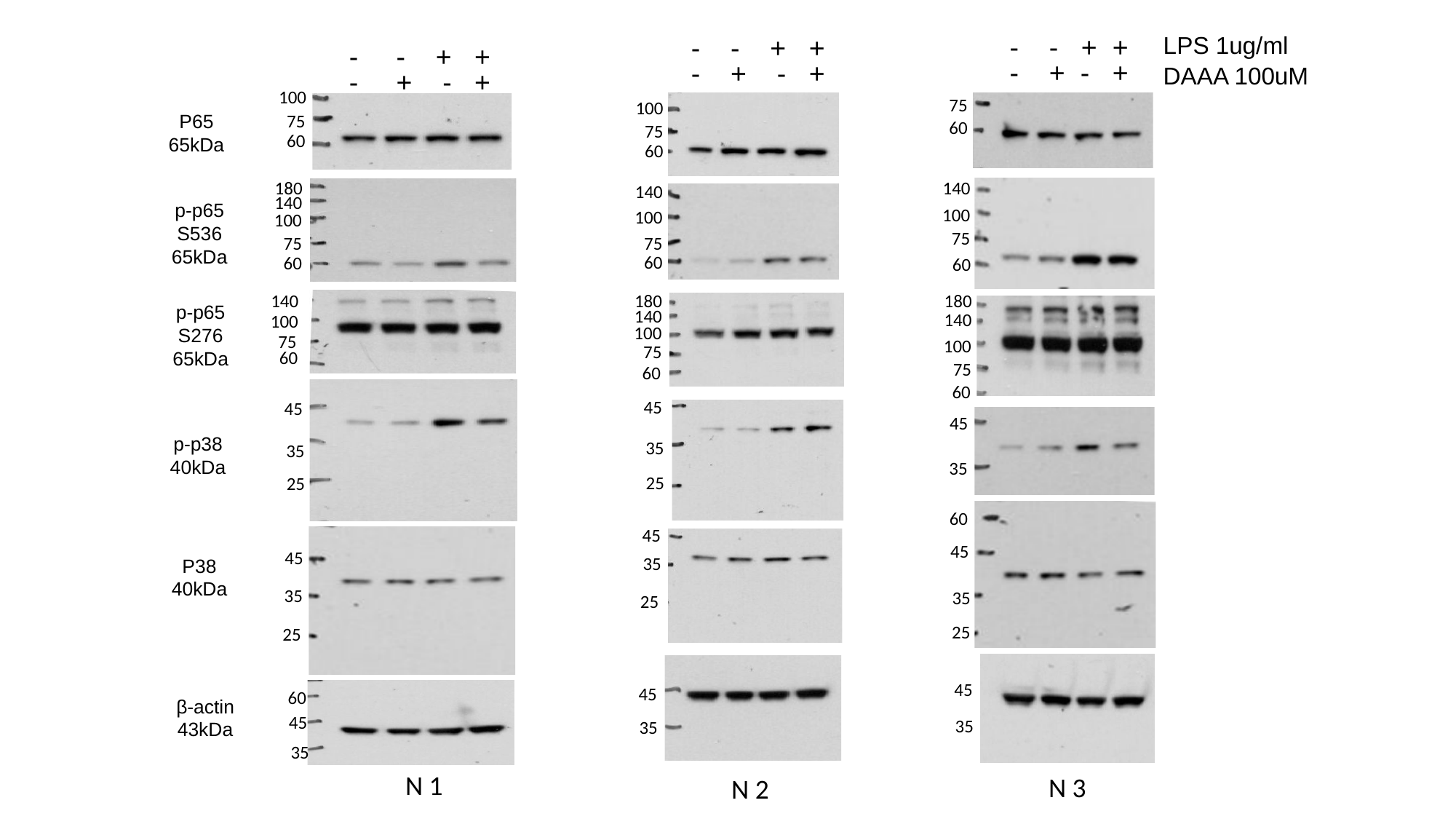

- - + +
LPS 1ug/ml
- - + +
- - + +
- + - +
- + - +
DAAA 100uM
- + - +
100
75
100
P65
65kDa
75
60
75
60
60
140
180
140
140
p-p65
S536
65kDa
100
100
100
75
75
75
60
60
60
180
180
140
p-p65 S276
65kDa
140
140
100
100
75
100
75
60
75
60
60
45
45
45
p-p38
40kDa
35
35
35
25
25
60
45
45
45
35
P38
40kDa
35
35
25
25
25
45
45
60
β-actin
43kDa
45
35
35
35
N 1
N 3
N 2
